# Rapid Identification of Officinal Akebiae Caulis and Its Toxic Adulterant Aristolochiae Manshuriensis Caulis (*Aristolochia manshuriensis*) by Loop-Mediated Isothermal Amplification

**DOI:** 10.3389/fpls.2016.00887

**Published:** 2016-06-20

**Authors:** Lan Wu, Bo Wang, Mingming Zhao, Wei Liu, Peng Zhang, Yuhua Shi, Chao Xiong, Ping Wang, Wei Sun, Shilin Chen

**Affiliations:** ^1^Key Laboratory of Beijing for Identification and Safety Evaluation of Chinese Medicine, Institute of Chinese Materia Medica, China Academy of Chinese Medical SciencesBeijing, China; ^2^College of Pharmacy, Hubei University of Chinese MedicineWuhan, China; ^3^Institute of Disease Control and Prevention, Academy of Military Medical SciencesBeijing, China

**Keywords:** loop-mediated isothermal amplification (LAMP), *Akebia*, *Aristolochia manshuriensis*, internal transcribed spacers 2 (ITS2), rapid authentication, visual detection

## Abstract

Mu-tong (Akebiae Caulis) is a traditional Chinese medicine commonly used as a diuretic and antiphlogistic. A common adulterant of Mu-tong is Guan-mu-tong (Aristolochiae Manshuriensis Caulis), which is derived from the stem of *Aristolochia manshuriensis* Komarov, and contains carcinogenic aristolochic acids. We used an alternative technique, loop-mediated isothermal amplification (LAMP), to differentiate Mu-tong from Guan-mu-tong because LAMP is quick, highly sensitive, and specific. We designed a set of four common primers (G-F3, G-B3, G-FIP, and G-BIP) and a loop primer (G-LB) for LAMP based on the internal transcribed spacer 2 sequence of *Ar. manshuriensis*. We successfully amplified the LAMP assays and visual detection occurred within 60 min at isothermal conditions of 65°C. The LAMP reaction exhibited a tenfold increase in detection (4.22 pg/μl DNA) over conventional polymerase chain reaction demonstrating that LAMP is a useful technique to detect Guan-mu-tong. We conclude that the LAMP technique is a potentially valuable safety control method for simple and efficient discrimination of Mu-tong from its adulterant Guan-mu-tong.

## Introduction

The use of traditional herbal medicine as dietary supplements has become increasingly popular in many countries due to the consumer’s belief that herbal medicine is both natural and harmless. In order to establish proper regulatory oversight for consumer safety, a procedure is needed that not only identifies the herbal plant in question, but also detects possible adulterants. One such case is that of Mu-tong (Akebiae Caulis), a common traditional Chinese medicine used as a diuretic and antiphlogistic (Kawata et al., 2007) which may be adulterated by an aristolochic acid containing herb, Guan-mu-tong (Aristolochiae Manshuriensis Caulis) derived from the stem of *Aristolochia manshuriensis* Komarov (Aristolochiaceae) (But and Ma, 1999; Lord et al., 1999; Ma and Zhang, 2002). Guan-mu-tong contains nephrotoxic and carcinogenic aristolochic acids (AAs), which cause aristolochic acid nephropathy (AAN) and upper tract urothelial carcinomas (UTUC) (Grollman et al., 2007; Hoang et al., 2013; Poon et al., 2013), with the inadvertent use of adulterated Guan-mu-tong by consumers leading to possible renal failure (But and Ma, 1999; Lord et al., 1999).

**FIGURE 1:**
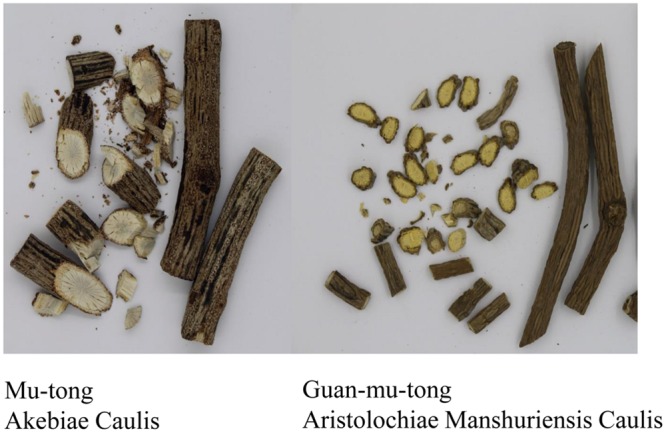
**Samples of Akebiae Caulis and Aristolochiae Manshuriensis Caulis**.

According to the Pharmacopoeia of the People’s Republic of China 2015 (State Pharmacopoeia Committee, 2015), Mu-tong comes from the stems of *Akebia quinata* (Houtt.) Decne., *Ak. trifoliata* (Thunb.) Koidz. and *Ak. trifoliata* (Thunb.) Koidz. var. *australis* (Diels) T. Shimizu. Not only is Mu-tong believed to have diuretic and anti-inflammatory properties, but it is also commonly used for weight loss and one species of Mu-tong (*Ak. quinata*) is an important ingredient used in a traditional Korean medicine. With seemingly similar common names, Mu-tong and Guan-mu-tong are commonly confused, and adding to the confusion is the post-harvest process, which makes proper identification of the dried stems difficult despite differing morphological characteristics of the plants (**Figure 1**). Currently, high performance liquid chromatography (HPLC) and HPLC–MS are able to differentiate between Aristolochiaceae plant material and non-Aristolochiaceae material through the analysis of aristolochic acid I, II, and other analogs (Hashimoto et al., 1999; Wei et al., 2005). However, both the HPLC and HPLC–MS methods may be hindered by climatic and altitudinal effects, which can cause chemical constituent changes. In another approach, DNA sequencing and DNA barcoding approaches are used for authentication of Aristolochiaceae and non-Aristolochiaceae plant material, but these procedures are time consuming (Han et al., 2005; Li et al., 2014; Wu et al., 2015). Due to these limitations, a rapid and effective identification method is needed for detecting Guan-mu-tong, in adulterated samples of Mu-tong.

Loop-mediated isothermal amplification (LAMP) is a quick, yet effective technique for amplifying DNA under isothermal conditions, which shows both high sensitivity and specificity. Due to LAMP’s simplicity, ease of use, prompt results, and straight-forward visual interpretation (Notomi et al., 2000; Mori et al., 2001; Nagamine et al., 2001; Mori and Notomi, 2009), this technique has been used for diagnosis of viral diseases (Mansour et al., 2015; Mongan et al., 2015; Nyan and Swinson, 2016; Venkatesan et al., 2016). In addition, LAMP has been used for the detection of: fungi (Niessen, 2015), milk pathogens (Cornelissen et al., 2016), tumors (Horibe et al., 2007), and recently herbal medicine adulterants. For example, the RT-LAMP assay was able to detect eastern or western strains of bluetongue virus in India in 60–90 min with the sensitivity equal to real-time RT-PCR but greater than conventional RT-PCR (Maan et al., 2016). In another example, the LAMP assay was used to rapidly and precisely discriminate the feces of sika deer (*Cervus nippon*) from Japanese serow (*Capricornis crispus*) within 60 min at 63°C (Aikawa et al., 2015). Furthermore, Bosward et al. (2016) reported a sensitivity of 20 pg of extracted DNA/reaction for a LAMP assay designed to detect *Streptococcus agalactiae* in bovine milk. Although previous studies have demonstrated the successful applicability of the LAMP technique in authenticating traditional Chinese medicinal materials such as: *Taraxacum formosanum* (Lai et al., 2015), *Catharanthus roseus* (Chaudhary et al., 2012), *Panax ginseng* (Sasaki et al., 2008), and *Hedyotis diffusa* (Li et al., 2013); to our knowledge, LAMP has not been applied for discriminating between Mu-tong and its toxic adulterant Guan-mu-tong. DNA barcoding is becoming a universal molecular technique used for effective species identification. Currently, due to high species resolution and moderate sequence length, the internal transcribed spacer 2 (ITS2) region has been widely used to authenticate plant species, especially medicinal plants (Lv et al., 2015; Xin et al., 2015; Zhao et al., 2015; Michel et al., 2016). Therefore, the ITS2 sequence is a suitable target-gene choice for designing primers for the LAMP assay. The aim of this study is to develop a sensitive and specific LAMP method based on the ITS2 sequence to rapidly and accurately identify Mu-tong and its toxic adulterant Guan-mu-tong.

## Materials and Methods

### Plant Materials

We collected three samples of each Mu-tong species (*Ak. quinata, Ak. trifoliata* and *Ak. trifoliata* var. *australis*) as well as three samples of the adulterant Guan-mu-tong (*Ar. manshuriensis*) for testing. The samples were collected from Jilin, Shanxi and Beijing (**Table 1**) and were identified by Junlin Yu at Tonghua Normal University, College of Pharmaceutical and Food Science and also Wei Sun at the Institute of Chinese Materia Medica, China Academy of Chinese Medical Sciences. All materials were stored in the Herbarium of the Institute of Medicinal Plant Development, Chinese Academy of Medical Sciences, Beijing, China.

**Table 1:** Descriptions of both Mu-tong and Guan-mu-tong samples used in the study.

Common Name	Latin name	Sample name	Source	Sample number
Mu-tong (Akebiae Caulis)	*Akebia trifoliata*	Mt-1, Mt-2, Mt-3	Jiangxi, Wuhan, Shanghai, China	3
Mu-tong (Akebiae Caulis)	*Ak. trifoliata* var. *australis*	Mt-4, Mt-5, Mt-6	Guangzhou, Yunan, China	3
Mu-tong (Akebiae Caulis)	*Ak. quinata*	Mt-7, Mt-8, Mt-9	Jiangxi, Henan, China	3
Guan-mu-tong (Aristolochiae Manshuriensis Caulis)	*Aristolochia manshuriensis*	Gmt-1, Gmt-2, Gmt-3	Jilin, Beijing, Shanxi, China	3

### DNA Isolation and Quantification

We used approximately 30 mg of fresh leaves or stems for DNA isolation utilizing the Plant Genomic DNA Kit (Tiangen Biotech Co., China) and we determined total DNA concentrations using Qubit^®^ 2.0 (Life Tech, Invitrogen, USA). We validated the identification of all plant material specimens using PCR-based sequences.

### Primer Design

**FIGURE 2:**
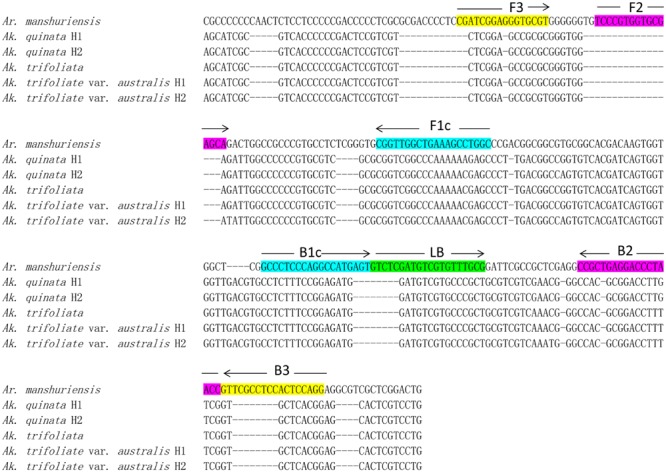
**Sequence alignment of the internal transcribed spacer 2 (ITS2) of Aristolochiae Manshuriensis Caulis and Akebiae Caulis.** Hyphens (-) denote alignment gaps. The specific primer and loop primer were designed from seven loci (F3, F2, FIc, B1c, B2, B3, and LB) as indicated in color boxes. Arrows represent the direction of 5′-3′ amplification in loop-mediated isothermal amplification (LAMP). H1 and H2 indicate different haplotypes.

DNA barcoding is a useful and effective tool, which enables the authentication of plant species accurately by using the ITS2 sequence, which is considered suitable for the identification of medicinal plants. We obtained ITS2 sequences of *Ar. manshuriensis, Ak. quinata, Ak. trifoliata* and *Ak. trifoliata* var. *australis* using DNA barcoding methods and based on these sequences, we designed LAMP primers for *Ar. manshuriensis*. The sequence alignment was carried out by MUSCLE in software MEGA 5.0. The variation of the ITS2 sequence within *Ar. manshuriensis, Ak. quinata, Ak. trifoliata* and *Ak. trifoliata* var. *australis* was aligned as illustrated in **Figure 2**. By utilizing PrimerExplorerV4^1^, we designed four common primers (G-F3, G-B3, G-FIP, and G-BIP) and a loop primer (G-LB) from ITS2 sequence of *Ar. manshuriensis* and are as follows: the forward outer primer G-F3 (5′-CGATCGGAGGGTGCGT-3′), backward outer primer G-B3 (5′-CCTGGAGTGGAGGCGAAC-3′), forward inner primers G-FIP (5′-GCCAGGCTTTCAGCCAACCGTCCCGTGGTGCGAGCA-3′), backward inner primer G-BIP (5′-GCCCTCCCAGGCCATGAGTGGTTAGGGTCCTCAGCGG-3′) and loop backward G-LB (5′-GTCTCGATGTCGTGTTTGCG-3′) (**Table 2**).

**Table 2:** Primers used in this study.

Assay	Primers	Sequence (5′to 3′)	Bases
LAMP	G-F3	CGATCGGAGGGTGCGT	16
	G-B 3	CCTGGAGTGGAGGCGAAC	18
	G-FIP (F1c&F2)	GCCAGGCTTTCAGCCAACCG-TCCCGTGGTGCGAGCA	36
	G-BIP (B1c&B2)	GCCCTCCCAGGCCATGAGT-GGTTAGGGTCCTCAGCGG	37
	G-LB	GTCTCGATGTCGTGTTTGCG	20
Conventional PCR	ITS2-2F^∗^	ATGCGATACTTGGTGTGAAT	20


	ITS2-3R^∗^	GACGCTTCTCCAGACTACAAT	21

*G-F3, forward outer primer; G-B3, backward outer primer; G-FIP, forward inner primers; G-BIP, backward inner primer; G-LB, loop backward.*

*^∗^Universal primers for ITS2 (Chen et al., 2010).*

### LAMP Reaction

All LAMP reactions occurred in a 25 μl mixture (DNA amplification kit; Loopamp, Eiken China Co., Ltd., Shanghai, China), which contained 2 × Reaction Mix, 1 μl *Bst* DNA polymerase, 0.2 μM of each outer primer (F3 and B3), 1.6 μM of each inner primer (FIP and BIP), 0.8 μM of loop primer (LB), 1 μl DNA template. We performed the LAMP reaction in an isothermal amplification system at 65°C for 60 min followed by inactivation at 80°C for 5 min. Additionally, EvaGreen fluorescent dye (Biotium, Hayward, CA) (20 × in water) was added to give a final concentration of 0.5×, when the LAMP reaction was carried out in a real-time PCR machine (Rotor-Gene Q, QIAGEN, Germany).

### Detection of LAMP Products

We utilized three different methods to detect LAMP products. First, we monitored real-time amplification of LAMP assays using a Loopamp real-time turbidimeter (LA-230; Eiken Chemical Co., Ltd., Tochigi, Japan) to monitor turbidity by recording the optical density at 400 nm every 6 s. Second, we employed visual inspection where we observed color changes in the LAMP reaction mixtures, caused by the addition of 1 μl of calcein (Fluorescence Detection Reagent; Loopamp, Eiken China Co., Ltd., Shanghai, China) before the LAMP reaction. Third, we monitored fluorescence of the EvaGreen dye in a Rotor-Gene Q. The reaction mixtures were conducted at 65°C for 60 min. Real-time fluorescence data was acquired every 60 s (a total of 60 cycles) on the Green channel (excitation at 470 nm and detection at 510 nm), followed by melting curve analysis from 65 to 99°C using 1°C steps. Real-time LAMP results were analyzed by *T*_t_ (time threshold) values.

### Specificity and Sensitivity of LAMP

The samples of *Ar. manshuriensis* (Guan-mu-tong) were used to assess the specificity of the LAMP test, which we conducted by preparing genomic DNA from *Ar. manshuriensis* in serial 10-fold dilutions to produce concentrations ranging from 42.2 ng/μl to 42.2 fg/μl. We amplified both LAMP and conventional PCR for sensitivity testing.

### Conventional PCR and Real Time PCR

We performed conventional PCR methodology by using a 25 μl mixture containing 12.5 μl Taq PCR Mix (Beijing Aidlab Biotech Co., China), 1 μl of each 2F and 3R primers (2.5μM) (**Table 2**), and 1 μl DNA template. We implemented the PCR reaction at 94°C for 5 min; followed by 40 cycles at 94°C for 30 s, 56°C for 30 s, and 72°C for 45 s; and a final extension step at 72°C for 10 min. We inspected the amplification products by 1% agarose gel electrophoresis. Furthermore, real-time PCR was carried out in Rotor-Gene Q (QIAGEN, Germany) by using 12.5 μL 2 × HRM PCR master mix (Type-it^®^ HRM^TM^ PCR Kit, QIAGEN, Germany). The reaction was performed as described above and followed by a melting analysis from 65 to 99°C with increment 1°C/s. We analyzed the real-time PCR results in terms of *T*_t_ (time threshold) values.

### Detection of Mixed Sample

To detect *Ar. manshuriensis* within a mixed sample, we combined *Ar. manshuriensis* and *Ak. quinata* plants into mixed proportioned samples which were then used for the contamination test. The proportioned samples of *Ar. manshuriensis* and *Ak. quinata* were mixed in the ratios of 1:99, 3:97, 5:95, 7:93, 10:90, 20:80, 30:70, 40:60, 50:50, 60:40, 70:30, 80:20, 90:10, and 99:1, respectively. We used DNA extraction to obtain the genomic DNA. After LAMP amplification, we observed results using visual inspection and real-time fluorescence curve.

## Results

### Specificity of LAMP

**FIGURE 3:**
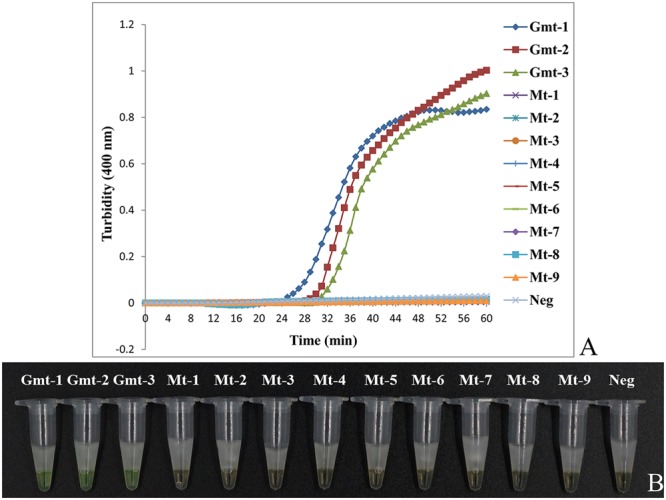
**Differentiation of Akebiae Caulis from Aristolochiae Manshuriensis Caulis using LAMP.** Gmt-1 to Gmt-3, *Aristolochia manshuriensis*; Mt-1 to Mt-3, *Akebia trifoliata*; Mt-4 to Mt-6, *Ak. trifoliata* var. *australis*; Mt-7 to Mt-9, *Ak. quinata*; Neg, negative control (double-distilled water). **(A)** Turbidity was monitored by a Loopamp real-time turbidimeter at 400 nm every 6 s, amplification was performed at 65°C for 60 min; **(B)** A visual color change detection method was compared. 1 μl of calcein (fluorescent detection reagent) was added to 25 μl of LAMP reaction mixture before the LAMP reaction.

To test the specificity of LAMP to detect Guan-mu-tong in adulterated samples of Mu-tong, we used the samples of *Ar. manshuriensis* as the positive samples, the three *Akebia* species as the negative samples and double-distilled water as the negative control. As depicted in **Figure 3A**, Guan-mu-tong (Gmt-1–Gmt-3) was detected by LAMP within 25–30 min and reached saturation at 45–60 min. In contrast, LAMP of the three *Akebia* species (Mt-1–Mt-9) and double-distilled water showed negative throughout the 60 min (**Figure 3A**) reaction, suggesting that the primer group could be used to detect Guan-mu-tong. We obtained similar results by directly observing the color change caused by the addition of calcein to the LAMP samples. Upon completion of the LAMP assay, positive reactions (Gmt-1–Gmt-3) changed from orange to green, whereas the negative reactions (Mt-1–Mt-9) and negative control (double-distilled water) remained orange (**Figure 3B**). Thus, these two detection methods showed the same sensitivity, indicating that the LAMP method developed in this study is able to promptly discriminate Guan-mu-tong from Mu-tong within 60 min under isothermal conditions.

### Sensitivity of LAMP

**FIGURE 4:**
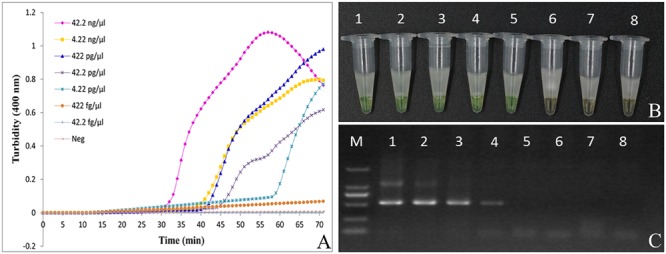
**Comparison of sensitivity between the LAMP reaction and conventional PCR for detection of Aristolochiae Manshuriensis Caulis.** The pure genomic DNA extracted from *Aristolochia manshuriensis* was diluted in a serial 10-fold dilution. Both LAMP reaction **(A)** and **(B)** and conventional PCR **(C)** were carried out in duplicate for each dilution point. Tubes and lanes: 1, 42.2 ng/μl; 2, 4.22 ng/μl; 3, 422 pg/μl; 4, 42.2 pg/μl; 5, 4.22 pg/μl; 6, 422 fg/μl; 7, 42.2 fg/μl; 8, Neg, negative control (double-distilled water). **(A)** Turbidity was monitored by a Loopamp real-time turbidimeter at 400 nm every 6 s; **(B)** A visual color change detection method was compared. 1μl of calcein (fluorescent detection reagent) was added to 25 μl of LAMP reaction mixture before the LAMP reaction; **(C)** The PCR products were detected by 1% agarose gel electrophoresis.

Our results show that the turbidity-based real-time LAMP assay of *Ar. manshuriensis* was detectable with 42.2 ng/μl to 4.22 pg/μl of DNA template (**Figure 4A**). Similarly, results from the direct visual method showed that the positive reactions (1–5) turned green, while the negative samples and negative control (6–8) remained orange (**Figure 4B**). We were able to confirm the sensitivity of the reactions by using the fluorescence-based real-time LAMP platform, with DNA templates ranging in concentration from 42.2 ng/μl to 4.22 pg/μl per reaction tube, and Tt values ranging from 17.28 to 43.51 min (Additional File: **Supplementary Figure S1A**). No amplification was acquired for the 422 and 42.2 fg/μl templates. Thus, the detection limit of the LAMP assay for authentication *Ar. manshuriensis* developed in this study was 4.22 pg/μl (**Figures 4A,B**; Additional File: **Supplementary Figure S1A**). For comparison purposes, we also performed conventional PCR using the universal primers for ITS2 and found that the detection limit for conventional PCR was 42.2 pg/μl (**Figure 4C**). We also found that in the real-time PCR platform, the Tt values increased from 33.16 to 45.99 min for templates ranging from 42.2 ng/μl to 42.2 pg/μl per reaction tube (Additional File: **Supplementary Figure S1B**). As to its sensitivity, however, no amplification was observed when the concentration of template ranged from 4.22 pg/μl to 42.2 fg/μl, indicating that the sensitivity is lower than that of LAMP. These results suggest that LAMP is a highly sensitive method for detection of *Ar. manshuriensis* genomic DNA.

### Detection of *Ar. manshuriensis* within a Mixed Sample

**FIGURE 5:**
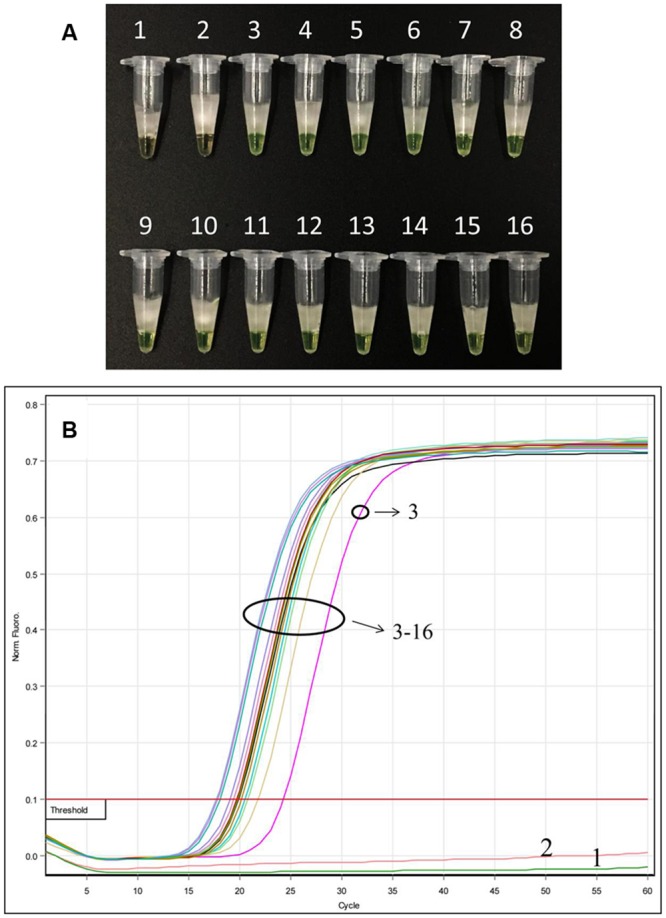
**Detection of *Ar. manshuriensis* within a mixed sample in various ratios using the LAMP method.** The sample powder of *Ar. manshuriensis* and *Ak. quinata* were mixed in the ratio 1:99, 3:97, 5:95, 7:93, 10:90, 20:80, 30:70, 40:60, 50:50, 60:40, 70:30, 80:20, 90:10, and 99:1, respectively. 1: *Ak. quinata* (negative sample); 2: double-distilled water (negative control); 3: 1:99; 4: 3:97; 5: 5:95; 6: 7:93; 7: 10:90; 8: 20:80; 9: 30:70; 10: 40:60; 11: 50:50; 12: 60:40; 13: 70:30; 14: 80:20; 15: 90:10; and 16: 99:1. **(A)** A visual color change detection method was compared. 1 μl of calcein (fluorescent detection reagent) was added to 25 μl of LAMP reaction mixture before the LAMP reaction; **(B)** In real time LAMP reaction, we monitored fluorescence in a Rotor-Gene Q.

After LAMP amplification, results from the direct visual method showed that all mixed samples in this study changed color from orange to green, indicating positive results in all mixed samples of different ratios (**Figure 5A**), with the exception of samples 1 and 2 (negative sample and double-distilled water, respectively), which remained orange. Additionally, we observed from the fluorescence-based real-time LAMP assay that the amplification curve appeared for all the mixed proportioned samples (**Figure 5B**); the Tt values increased from 17.72 to 24.30 min for all mixed ratios (1:99 to 99:1) of *Ar. manshuriensis* (**Figure 5B**). No amplification curve was obtained from either the negative sample or the double-distilled water (samples 1 and 2). These results demonstrate that *Ar. manshuriensis* within a mixed sample can be detected using the LAMP method when its ratio greater than or equal to 1% in the mixed sample. As a result, the developed LAMP method for *Ar. manshuriensis* detection is prompt and reliable for situations where Mu-tong may be contaminated by Guan-mu-tong.

## Discussion

In this study, we investigated the usefulness of the LAMP method as a prompt and effective way of detecting Guan-mu-tong, as a common adulterant of Mu-tong. Currently, few methods are available for the identification Akebiae Caulis and its toxic adulterant Aristolochiae Manshuriensis Caulis (*Ar. manshuriensis*). Some previous studies have shown that both the PCR-based assay (Han et al., 2005; Li et al., 2014; Wu et al., 2015) and chemical method (Hashimoto et al., 1999; Wei et al., 2005) can also be used to authenticate Akebiae Caulis and Aristolochiae Manshuriensis Caulis. While these methods are able to accurately discriminate between species, they require complicated and time-consuming procedures, delicate instruments, and professional statistical methodologies.

The basic principles of PCR-based assay are: DNA fragment amplification and fragment detection via agarose gel electrophoresis. During conventional PCR procedures, at least three steps have high probabilities of introducing undesirable effects such as denaturation, annealing, and extension. In general, to conduct a PCR assay it takes approximately 2–3 h, depending on parameters, but this timeframe may be considered too time consuming for some situations. In contrast, the LAMP assay is performed in isothermal conditions (60–65°C) because LAMP requires *Bst* DNA polymerase, which has unique strand displacement activity. This characteristic allows not only for the LAMP reactions to occur faster, but also reduces the possibility of denaturation during the procedure. For example, in this study, we show that the developed LAMP method can detect *Ar. manshuriensis* from Akebiae Caulis at 65°C within 60 min. In evaluating the detection of Guan-mu-tong from mixed samples, we show that contamination can be detected at 65°C within 60 min given that the ratio of the contaminant, *Ar. manshuriensis* is at least 1%. The simplicity of the procedure suggests that it will be a significant, useful, and convenient method for identification of toxic herbal contaminants from tainted nontoxic herbal plant samples.

Moreover, PCR requires high-precision instrumentation due to the temperature cycling environment needed, whereas LAMP can be performed in constant temperature conditions decreasing the need for expensive equipment. Other detection procedures such as chemical detection (i.e., U/HPLC and U/HPLC–MS) also require highly specialized technology such as chromatographic instruments. In contrast, the LAMP method needs no such expensive instrumentation; only a water bath or heating block is enough. Additionally, agarose gel electrophoresis is required after amplification in conventional PCR to determine results, but the LAMP results can be detected by several methods. These detection methods include a simplified methodology that allows an individual to obtain LAMP results by use of the naked eye when an additional metal indicator such as calcein (Parida et al., 2008; Tomita et al., 2008), hydroxy naphthol blue (HNB) (Goto et al., 2009), or dye SYBR green (Parida et al., 2005) is added to the sample. Where SYBR green is added after LAMP amplification; calcein or HNB can be added at the beginning of the reaction to reduce the chance of contamination. A second method of detecting LAMP amplification products can be accomplished by monitoring with a real-time turbidimeter. Lastly, LAMP results are also detectable through agarose gel electrophoresis, similar to conventional PCR procedures, but we suggest avoiding this method because open-tube operation increases the risk of contamination.

Despite the many advantages of the LAMP reaction, several drawbacks to this procedure do exist. First, we acknowledge the occurrence of false positives due to high amplification efficiency as well as contamination issues. To address contamination during the LAMP procedure, we added low-melting-point paraffin wax to the reaction tubes to prevent contaminants, which previous studies show to be effective for the LAMP technique (Li et al., 2012; Liu et al., 2012). In addition, spatial separation of the reagent and test samples is needed in order to reduce the risk of contamination. Second, frequently used barcodes are not always suitable for designing LAMP primers. The primer design is an important step in the LAMP procedure and selecting a proper barcode marker is the basis of the primer design. The barcode sequence must be able to distinguish species effectively and include all regions adapted for the design of LAMP primers. Thus, we suggest try to design some groups of LAMP primers as candidates based on common barcode markers like ITS2, *matK*, or *psbA-trnH* etc. The best group of primers can be obtained by screening candidate primers in amplification efficiency.

The application of DNA barcoding in the identification of herbal materials is widely used, with ITS2 proposed as a core marker and *psbA-trnH* as a supplementary marker for differentiation of medicinal plants (Chen et al., 2010, 2014; Li et al., 2015). The wide use of the ITS2 sequence is due in great part to the successful identification of medicinal plant material by previous studies (Gu et al., 2013; Xin et al., 2013, 2015; Zhao et al., 2015) as well as its easy amplification. The use of the *psbA-trnH* sequence is less effective than ITS2 as shown both by our previous work with Aristolochiaceous plant materials (Wu et al., 2015), as well as this current study, which found that the *psbA-trnH* sequence is not suitable for obtaining LAMP primers. No primers were obtained when we uploaded the *psbA-trnH* sequence to PrimerExplorerV4 to design a LAMP primer. However, two sets of LAMP primers (each containing four primers: FIP, BIP, F3, and B3) were obtained from PrimerExplorerV4^2^ based on the ITS2 sequence of *Ar. manshuriensis*. After we acquired the two LAMP primer sets, we tested both for usability and determined that only one set was useable (data not shown). In this study, the design of LAMP primers was based on the ITS2 sequence, which is a useful DNA barcode in *Aristolochia* species as well as its adulterant (Wu et al., 2015). Four primers (G-F3, G-FIP, G-BIP, and G-B3) and one loop primer (G-LB) were designed from seven loci in the ITS2 sequence of *Ar. manshuriensis* (**Figure 2**) which were specific to Guan-mu-tong (*Ar. manshuriensis*), but not the three *Akebia* (Mu-tong) species (**Figure 2**). The LAMP technique is a highly sensitive method, with DNA template amplification at low concentrations (six copies) (Notomi et al., 2000). We found that the LAMP assay for *Ar. manshuriensis* detection was tenfold more sensitive than conventional PCR assay sensitivity.

Although several methods were introduced to identify Akebiae Caulis and Aristolochiae Manshuriensis in previous studies, none of them could: (i) provide an on-site and rapid method for exact and effective authentication, nor (ii) provide results using simple, readily available heating devices. Compared to the several methods used to authenticate Akebiae Caulis from Aristolochiae Manshuriensis, results indicate that the LAMP assay is a superior process. Our study introduces a novel method for the detection of toxic Guan-mu-tong from widely used Mu-tong. With the increase of widespread attention of governmental agencies on the safety of traditional medicines, our results indicate that the LAMP method is an effective technique to test for the safety and quality of medicinal plant specimens due to its simplicity, rapidness, cost-effectiveness, and high sensitivity.

## Author Contributions

LW and BW performed the experiments. MZ collected and analyzed data. LW wrote the manuscript. WS, PW, WL, PZ, YS, CX and SC conceived the study design and read and approved the final manuscript.

## Conflict of Interest Statement

The authors declare that the research was conducted in the absence of any commercial or financial relationships that could be construed as a potential conflict of interest.

## Footnotes

1 http://primerexplorer.jp/elamp4.0.0/index.html

2 http://primerexplorer.jp/elamp4.0.0/index.html

## Supplementary Material

FIGURE S1 **Comparison of sensitivity between fluorescence-based real-time LAMP reaction and real-time PCR for detection of Aristolochiae Manshuriensis Caulis.** The pure genomic DNA extracted from *Aristolochia manshuriensis* was diluted in a serial 10-fold dilution. Both real time LAMP **(A)** and real time PCR **(B)** were carried out in duplicate for each dilution point. Tubes and lanes: 1, 42.2 ng/μl; 2, 4.22 ng/μl; 3, 422 pg/μl; 4, 42.2 pg/μl; 5, 4.22 pg/μl. **(A)** In real time LAMP reaction, we monitored fluorescence in a Rotor-Gene Q; **(B)** In real time PCR reaction, we monitored fluorescence in a Rotor-Gene Q.
