# Supplementary figures and images for: Rapid Identification of Officinal Akebiae Caulis and Its Toxic Adulterant Aristolochiae Manshuriensis Caulis (Aristolochia manshuriensis) by Loop-Mediated Isothermal Amplification
Source: Front Plant Sci. 2016 Jun 20;7:887. doi: 10.3389/fpls.2016.00887 (PMC4913086; doi:10.3389/fpls.2016.00887)

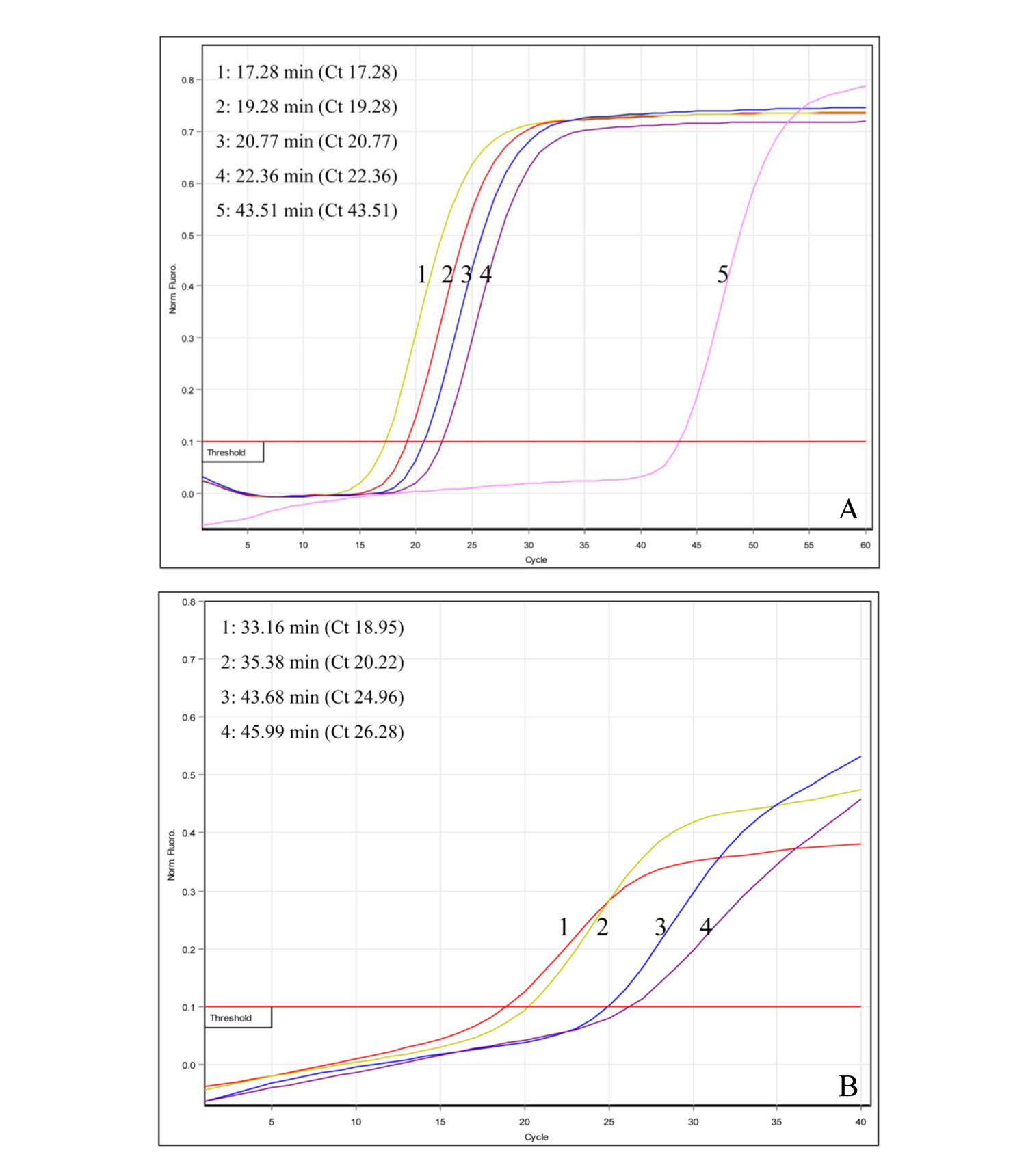

Supplement: FIGURE S1 — Comparison of sensitivity between fluorescence-based real-time LAMP reaction and real-time PCR for detection of Aristolochiae Manshuriensis Caulis. The pure genomic DNA extracted from Aristolochia manshuriensis was diluted in a serial 10-fold dilution. Both real time LAMP (A) and real time PCR (B) were carried out in duplicate for each dilution point. Tubes and lanes: 1, 42.2 ng/μl; 2, 4.22 ng/μl; 3, 422 pg/μl; 4, 42.2 pg/μl; 5, 4.22 pg/μl. (A) In real time LAMP reaction, we monitored fluorescence in a Rotor-Gene Q; (B) In real time PCR reaction, we monitored fluorescence in a Rotor-Gene Q. [file Image_1.TIF]
